# Antidepressant-like Effects of Combined Fluoxetine and Zinc Treatment in Mice Exposed to Chronic Restraint Stress Are Related to Modulation of Histone Deacetylase

**DOI:** 10.3390/molecules27010022

**Published:** 2021-12-21

**Authors:** Paulina Misztak, Magdalena Sowa-Kućma, Patrycja Pańczyszyn-Trzewik, Bernadeta Szewczyk, Gabriel Nowak

**Affiliations:** 1 Department of Pharmacobiology, Jagiellonian University Medical College, Medyczna 9, 30-668 Krakow, Poland; nowak@if-pan.krakow.pl (G.N.); 2 Department of Neurobiology, Maj Institute of Pharmacology, Polish Academy of Sciences, Smetna 12, 31-343 Krakow, Poland; 3 Department of Human Physiology, Institute of Medical Sciences, Medical College of Rzeszow University, Kopisto 2a, 35-959 Rzeszow, Poland; msowa@ur.edu.pl (M.S.-K.); ppanczyszyn@ur.edu.pl (P.P.-T.)

**Keywords:** zinc, fluoxetine, chronic restraint stress, an animal model of depression, antidepressants

## Abstract

Chronic stress is the key factor contributing to the development of depressive symptoms. Chronic restraint stress (CRS) is well validated and is one of the most commonly used models to induce depressive-like behavior in rodents. The present study aimed to evaluate whether fluoxetine (FLU 5 mg/kg) and zinc (Zn 10mg/kg) given simultaneously induce a more pronounced antidepressant-like effect in the CRS model than both those compounds given alone. Behavioral assessment was performed using the tail suspension and splash tests (TST and ST, respectively). Furthermore, the effects of CRS, FLU and Zn given alone and combined treatment with FLU + Zn on the expression of proteins involved in the apoptotic, inflammatory, and epigenetic processes were evaluated in selected brain structures (prefrontal cortex, PFC; and hippocampus, Hp) using Western blot analysis or enzyme-linked immunosorbent assays (ELISA). The results obtained indicated that three hours (per day) of immobilization for 4 weeks induced prominent depressive symptoms that manifested as increased immobility time in the TST, as well as decreased number and grooming time in the ST. Behavioral changes induced by CRS were reversed by both FLU (5 and 10 mg/kg) or Zn (10 mg/kg). Zinc supplementation (10 mg/kg) slightly increases the effectiveness of FLU (5 mg/kg) in the TST. However, it significantly increased the activity of FLU in the ST compared to the effect induced by FLU and Zn alone. Biochemical studies revealed that neither CRS nor FLU and Zn given alone or in combined treatment alter the expression of proteins involved in apoptotic or inflammatory processes. CRS induced major alterations in histone deacetylase (HDAC) levels by increasing the level of HADC1 and decreasing the level of HADC4 in the PFC and Hp, decreasing the level of HADC6 in the PFC but increasing it in Hp. Interestingly, FLU + Zn treatment reversed CRS-induced changes in HDAC levels in the Hp, indicating that HDAC modulation is linked to FLU + Zn treatment and this effect is structure-specific.

## 1. Introduction

According to the latest global statistics and the World Health Organization (WHO) report, major depressive disorder (MDD) is the most common mental disorder worldwide. It is characterized by mood and sleep disturbances, lack of interest and concentration, feeling of guilt, and suicidal ideation [1]. MDD pharmacotherapy is based on limited knowledge of its pathomechanism (primarily based on assumptions of monoaminergic theory), which is best has relatively low efficacy (only 30–45% of patients achieve remission) [2].

It is proven that chronic stress is the most critical factor contributing to the development of suicidal and depressive-like behaviors [3,4,5]. Stress, a state of impaired homeostasis results in many physiological disturbances and pathological processes depending on the severity, type, and duration of the stressor [6]. One of the consequences of stress in MDD patients is the activation of inflammatory and oxidative pathways, evidenced by the increase in pro-inflammatory cytokines, such as IL-1β, TNF-α, IL-6 or C-reactive protein, and markers of oxidative stress (respectively) in MDD patients [4,7,8,9,10]. It is thus not surprising that the best animal models of depression used to study the etiology of depression and search for new and more effective antidepressants are based on stress-induced alterations. Among those described in the literature, the most widely used are chronic mild stress (CMS), chronic restraint stress (CRS), chronic social defeat stress (CSDS), chronic unpredictable mild stress (CUMS), chronic unpredictable stress (CUS), social defeat stress (SDS) and the maternal separation (MS) model [11,12,13]. Interestingly, despite methodological differences (various stressors), most models are characterized by the development of similar symptoms, such as anxiety traits/fear or anhedonic behavior, disruption in learning, and hyperactivation of the hypothalamic–pituitary–adrenal (HPA) axis [14].

Chronic restraint stress (CRS) is well validated and one of the most commonly used models to induce depressive behavior in rodents [15,16]. Repeated immobilization results in several depression-like features, including anhedonia or anxiety behavior [17]. Mice subjected to the 6-h stress session for 28 days exhibited anxiety-/depression-like behavior and also memory and learning disabilities [18]. Furthermore, acute restraint stress (2 or 24 h) caused anxiety behavior revealed in the elevated plus-maze test (EPM) and open field test, as well as depressive/anhedonic behavior measured by the forced swim test (FST), contextual fear conditioning (CFC) and sucrose preference test (SPT) [19,20,21]. Ihne et al. demonstrated that mice subjected to CRS (2 h/day; 10 days) induced depressive-like behavior observed as increased immobility time in the FST [22]. Behavioral abnormalities induced by CRS are reversed by some antidepressants [17]. Christiansen et al. showed that mice subjected to 6 h of immobilization once daily for 14 days exhibited an increased immobility time in the TST, while fluoxetine prevented the development of CRS-induced behavioral disturbances [23]. Furthermore, after sub-chronic fluoxetine treatment (in drinking water), CRS-induced changes were reversed (Ihne et al., 2012).

Depressive disorders and stress-related diseases are characterized by the induction of oxidative and/or nitrosative stress and activation of inflammatory, and apoptotic pathways [8,24]. It is known that after exposure to stress, levels of pro-inflammatory cytokines such as IL-6, IL-1β, or TNF-α rise in peripheral blood, as well as in the brain [25]. A large body of evidence confirms that restraint stress increases blood levels of TNF-α, IL-6, and NF-κB, p38, and JNK (Jun N-terminal kinase) in PFC [18,26].

Recently, several studies have linked epigenetics and depression. Epigenetic mechanisms regulate the expression of genes but not their structure. One of the most important epigenetic changes is histone modification (acetylation/ deacetylation of histones). HDACs are enzymes that remove acetyl groups from ε-amino lysines of histone proteins to control gene transcription [27]. Several animal models of depression exhibit a disruption in HDACs’ activity. In the corticosterone (CORT) model of depression, HDAC2 was significantly elevated, while fluoxetine and vorinostat normalized their levels [28]. In maternal deprivation, HDACs’ activity was increased in the Hp and nucleus accumbens but quetiapine administration reversed these changes [29]. Another study revealed that the offspring of gestational stress dams exhibited depressive and anxiety-like behaviors accompanied by increased expression of HDAC1 and HDAC2 in Hp [30]. Recent data suggested the potent role of HDAC inhibitors in neurodegenerative and psychiatric disorders [31,32]. Vorinostat (SAHA), which inhibits the activity of histone deacetylases HDAC 1–3 and HDAC6, modulates inflammation and oxidative stress by reducing the level of malondialdehyde (MDA) and IL-1ß and the expression of the mRNA of the genes Nfkb1 and Hdac2 in the CORT model of depression [28]. On the other hand, HDAC4 overexpression is associated with the protection of the mouse brain against apoptosis and stress of the endoplasmic reticulum (ER) [33].

Several clinical studies have shown a relationship between dietary zinc deficiency and depression. Clinical studies also suggest the potential benefits of zinc supplementation as an adjunct to antidepressant therapy or as a stand-alone therapy for the prevention of depressive symptoms [34]. The beneficial effects of zinc treatment were also reported in preclinical studies. Zinc induced an antidepressant-like effect (reduction in immobility time) in both the FST and TST [35]. Zinc was also active in different models of depression: olfactory bulbectomy (OB) [36]; CMS [37]; CUS [38]. Furthermore, zinc was found to improve the effects of standard antidepressants (imipramine, fluoxetine, paroxetine, bupropion, or citalopram) on TST in mice; FST and CUS in rats [see [35,39]].

Based on these data, we decided to further evaluate the role of CRS in the induction of depressive-like behavior, proapoptotic and inflammatory processes, and epigenetic changes. Furthermore, Zn supplementation and FLU in the reversion of CRS-induced depressive-like alterations were evaluated. Finally, we decided to check whether zinc supplementation will increase the antidepressant effect of FLU in the CRS model and what mechanisms this combination may trigger.

## 2. Results

### 2.1. Establishing the Chronic Restraint Stress (CRS) Protocol Behavioral Studies

**Figure 1:**
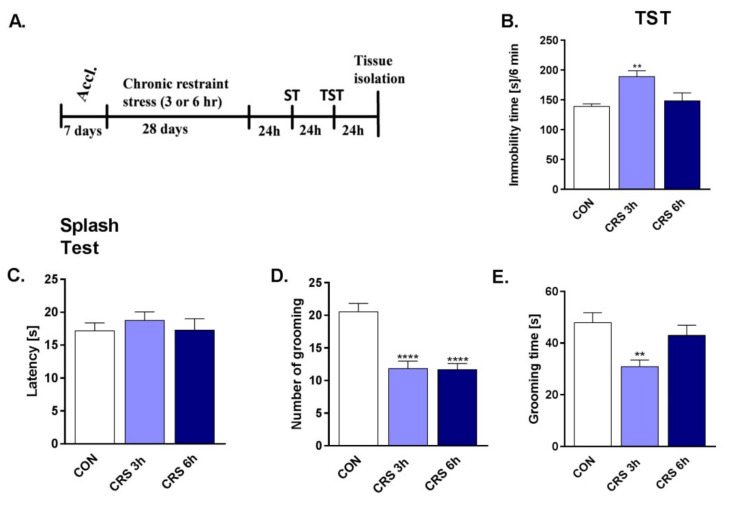
The effect of 3 and 6 h of immobility/day/28 days on the behavior of mice. (**A**) Experimental schedule; (**B**) Effects of CRS in the TST; (**C**–**E**) Effects of CRS in the ST in mice. Data were analyzed using one-way analysis of variance (ANOVA) and Dunnett’s multiple comparison test. Values are expressed as mean ± SEM. *n* = 19–22., ** *p* < 0.01, **** *p* < 0.0001 vs. CON. CON–control group; CRS-chronic restraint stress. Accl.-an acclimation period.

The first aim of this study was to establish a standardized protocol for CRS. Therefore, we investigated the induction of depressive effects of CRS in mice using 3- and 6-h daily immobility for 28 days. For this, the tail suspension test (TST) and the splash test (ST) were used to measure the depressive-like effects of CRS. The experimental schedule is shown in Figure 1A.

The results obtained indicate that 3 but not 6 h of immobilization/day/28 days increased the immobility time in TST in mice (*p* < 0.05), exhibiting its depressive-like effects (Figure 1B). In the ST (Figure 1C–E), 3 h of immobilization did not reduce latency time (*p* > 0.05) however decrease in grooming number (*p* < 0.001), and grooming time (*p* < 0.0001) was observed. In comparison, 6-hr daily immobility for 28 days decreased the number of grooming (*p* < 0.001) but did not influence the latency and grooming time (*p* > 0.05), confirming 3-h as the ideal time for the induction of depressive-like effects in the CRS model in mice.

Results of the one-way ANOVA analysis: TST: F (2.58) = 7882, *p* = 0.0009; ST: (C) F (2.58) = 0.3849, *p* = 0.6822; (D) F (2.58) = 22.43, *p* < 0.0001; (E) F (2.58) = 6312, *p* = 0.0033.

### 2.2. Effect of FLU Treatment on the CRS Model in Mice

**Figure 2:**
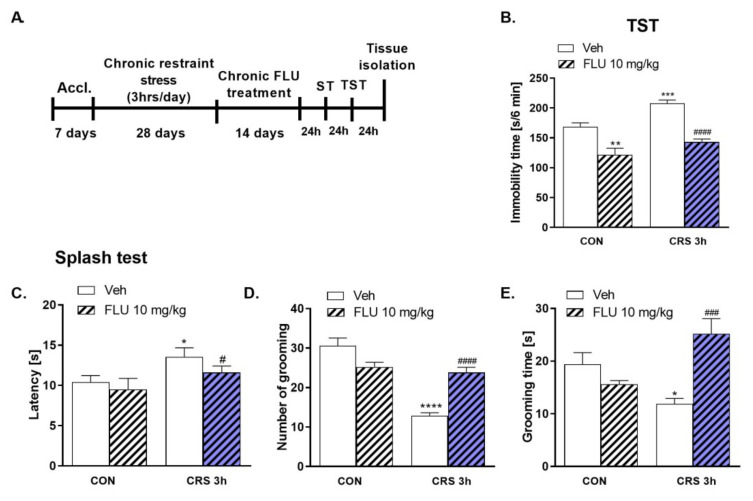
The effect of fluoxetine treatment on CRS-induced behavioral changes. (**A**) Experimental schedule; (**B**) Effects of CRS and FLU in the TST; (**C**–**E**) Effects of CRS and FLU in the ST in mice. All data were analyzed by two-way ANOVA and Newman–Keuls multiple comparison test. Values are expressed as mean ± SEM; *n* = 8–9. * *p* < 0.05, ** *p* < 0.01, *** *p* < 0.001, **** *p* < 0.0001 vs. CON, # *p* < 0.05, #### *p* < 0.0001 vs. CRS. CON—control group; CRS-chronic restraint stress; Accl.—an acclimation period.

Chronic treatment with FLU at a dose of 10 mg/kg was effective in reversing the CRS-induced increase in immobility time in the TST in mice (Figure 2B, *p* < 0.001). Moreover, FLU was found to normalize CRS-induced behavioral alterations in the ST (Figure 2C–E) such as decreased latency to grooming (*p* < 0.05), and increased number (*p* < 0.0001) and time of grooming (*p* < 0.001). The experimental schedule is shown in Figure 2A. Two- way ANOVA analysis: (B) TST: Effect of treatment F (1.35) = 57,31, *p* < 0.0001; effect of stress F (1.35) = 16,78, *p* = 0.0002; no interaction F (1.35) = 1455, *p* = 0.2358; ST: (C) latency: no effect of treatment F (1.35) = 1839, *p* = 0.1838; effect of stress F (1.35) = 6229 *p* = 0.0174; no interaction F (1.35) = 0.2513; *p* = 0.6193; (D) number of grooming: effect of treatment F (1.35) = 3834, *p* = 0.0582; effect of stress F (1.35) = 46.10, *p* < 0.0001; interaction F (1.35) = 33.58, *p* < 0.0001; € grooming time: effect of treatment F (1.35) = 5927, *p* = 0.0201; no effect of stress F (1.35) = 0.2859, *p* = 0.5962; interaction F (1.35) = 19.18, *p* = 0.0001.

### 2.3. Supplementation with Zn and FLU Treatment in the CRS Model in Mice–Antidepressant Effects

**Figure 3:**
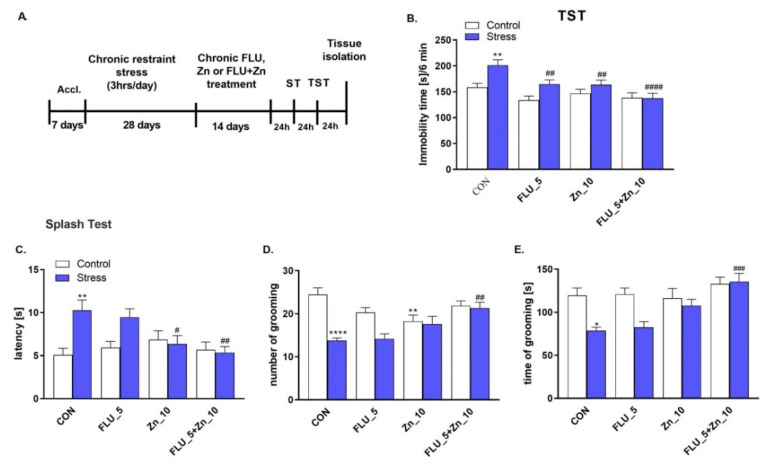
The effect of combined administration of fluoxetine (FLU; 5 mg/kg) and zinc (Zn; 10 mg/kg) on CRS-induced behavioral disturbances. (**A**) Experimental schedule; (**B**) Effects of CRS, FLU and Zn in the TST; (**C**–**E**) Effects of CRS, FLU and Zn in the ST in mice. Data were analyzed by two-way ANOVA and Newman–Keuls multiple comparison test. Values are expressed as mean ± SEM; *n* = 11–16. * *p* < 0.05 vs. CON, ** *p* < 0.01 vs. CON, **** *p* < 0.0001 vs. CON, # *p* < 0.05 vs. Stress, ## *p* < 0.01 vs. Stress, ### *p* < 0.001 vs. Stress, #### *p* < 0.0001 vs. Stress. CON—control group; Accl.—an acclimation period.

The main part of this work was to investigate whether Zn supplementation enhances the effect of FLU therapy in normalizing depressive-like behavior in the CRS model and describe the mechanisms underlying these effects. Therefore, the effect of chronic (14 days) zinc supplementation (10 mg/kg) to FLU (5 mg/kg) treatment in the CRS model (3 h immobilization/day /28 days) was studied. FLU (5 mg/kg) and zinc (10 mg/kg) given alone decreased the immobility time in the TST in mice subjected to CRS (*p* < 0.01), indicating antidepressant activity of each treatment (Figure 3B). However, the combined treatment of FLU + Zn induced a stronger effect in TST (*p* < 0.0001). A significant positive effect of Zn supplementation was evident in the ST (Figure 3C–E). Treatment with FLU + Zn reversed the CRS induced behavioral changes observed as decreased latency time to grooming (*p* < 0.01) and an increased number (*p* < 0.01), and grooming time of grooming (*p* < 0.001) (Figure 3C). The experimental schedule is shown in Figure 3A. The two-way ANOVA showed: TST (B): effect of stress: F (1100) = 13.02, *p* = 0.0005; effect of treatment: F (3100) = 0.0002; but not significant interaction: F (3100) = 2.099, *p* = 1.051; Splash test: (C) latency: effect of stress F (1100) = 9.121, *p* = 0.0032, no effect of treatment F (3100) = 2.491, *p* = 0.0645; and significant interaction F (3100) = 4.550, *p* = 0.0050; (D) number of grooming: effect of stress F (1100) = 21.87, *p* < 0.0001, effect of treatment F (3100) = 3.994, *p* = 0.0099; and significant interaction F (3100) = 6.343, *p* = 0.0006. € time of grooming: effect of stress F (1100) = 12.99, *p* = 0.0005, effect of treatment F (3100) = 7.238, *p* = 0.0002; and significant interaction effect F (3100) = 3.363, *p* = 0.0217.

### 2.4. Effect of CRS, FLU, and FLU + Zn Treatment on the Spontaneous Activity of Mice.

To eliminate false positive results in TST, the effects of the CRS procedure and the compounds studied, the general locomotor activity of the mice were measured (Table 1). There were no changes in locomotor activity of mice subjected to CRS (3/6 h/day/28 days) only (A, B, C) FLU (5, 10 mg/kg) alone (B,C); Zn (10 mg/kg) alone, and FLU (5 mg/kg) + Zn (10 mg/kg). One way ANOVA showed F = 1.736, *p*= 0.1493 Two-way ANOVA showed: (B) no stress effect [F (2.60) = 1.463; *p* = 0.2396], treatment effect [F (1.60) = 4.823; *p* = 0.0320] and no effect of interaction [F (2.60) = 2.827; *p* = 0.0671]; (C) stress effect [F (1101) = 7.331; *p* = 0.0080], no treatment effect [F (3101) = 2.503; *p* = 0.0635] and effect of interaction [F (3101) = 4.686; *p* = 0.0042].

### 2.5. Supplementation with Zn and Treatment with FLU in the CRS Model in Mice–Effect on the Level of BDNF Protein

**Figure 4:**
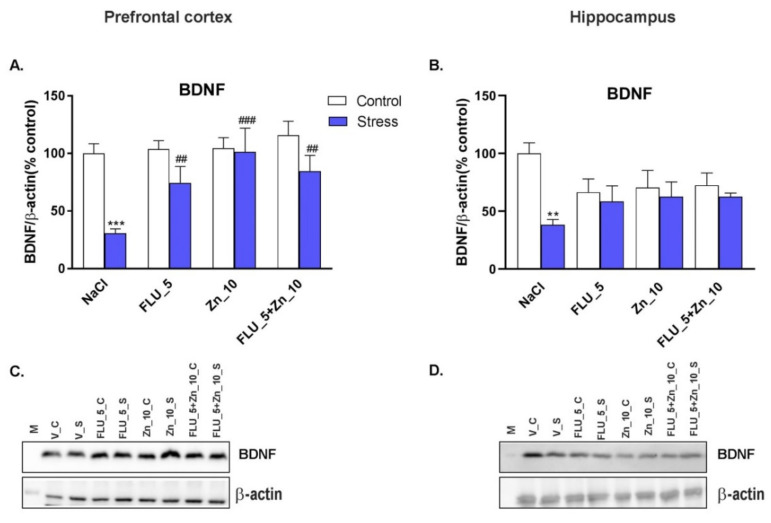
Effect of Zn supplementation and FLU treatment on the level of BDNF protein in PFC (**A**, **C**) and Hp (**B**, **D**) mice subjected to CRS. All data were analyzed by two-way ANOVA and Newman–Keuls multiple comparison test. Values are expressed as mean ± SEM; *n* = 7–8; *p* = 0.0260]. ** *p* < 0.01, *** *p* < 0.001 vs. NaCl, ## *p* < 0.01, ### *p* < 0.001 vs. CRS + NaCl.

CRS induced a significant decrease in BDNF protein levels in the PFC (Figure 4A) and Hp (Figure 4B) of mice (*p* < 0.001 and *p* < 0.01, respectively). The CRS induced decrease in BDNF protein level was reversed by FLU (5 mg/kg), Zn (10 mg/kg), and combined FLU + Zn chronic treatment (*p* < 0.05, *p* < 0.01, *p* < 0.05, respectively). Representative western blots are shown in Figure 4C,D. Two-way ANOVA showed: BDNF in the PFC: stress effect [F (1.51) = 16.93; *p* = 0.0001], treatment effect [F (3.51) = 4.831; *p* = 0.0049], interaction [F (3.51) = 3.076; *p* = 0.0357]; in Hp: stress effect [F (1.51) = 7.584; *p* = 0.0081], no treatment effect [F (3.51) = 0.0509; *p* = 0.9847] and effect of interaction [F (3.51) = 3.684, *p* = 0.0177.

### 2.6. Zinc Supplementation and FLU Treatment in the CRS Model in Mice-Effect on Pro-and Anti-Apoptotic Protein Levels.

**Figure 5:**
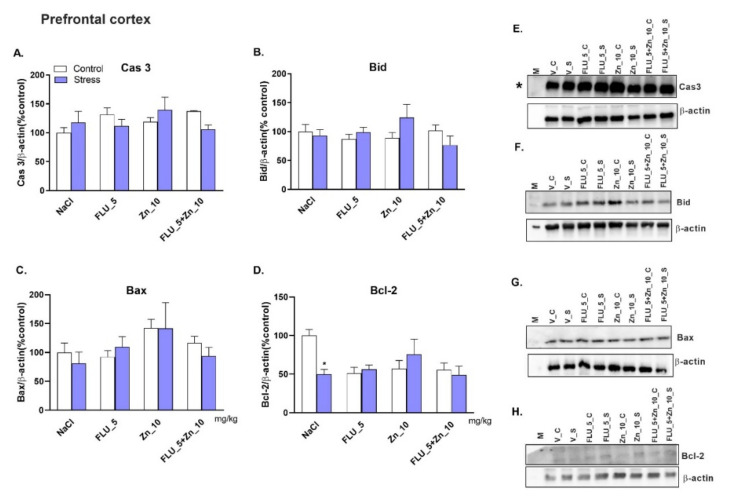
The effect of Zn supplementation and FLU treatment on caspase 3 (Cas3; **A**), Bid (**B**), Bax (**C**), and Bcl-2 (**D**) proteins in the PFC of mice subjected to CRS. All data were analyzed by two-way ANOVA and Newman–Keuls multiple comparison test. Values are expressed as mean ± SEM; *n* = 4–7. *p < 0.05 vs NaCl. (**E**–**H**) Representative western blots * images from the same membrane (please see Figure 8).

**Figure 6:**
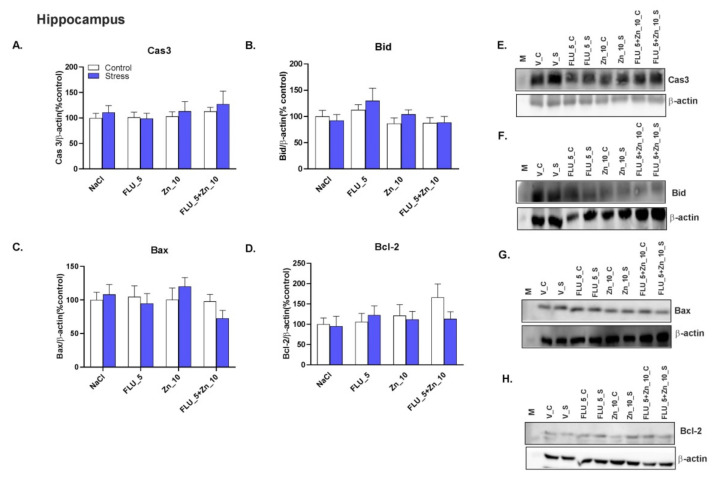
The effect of Zn supplementation and FLU treatment on caspase 3 (Cas3; **A**), Bid (**B**), Bax (**C**), and Bcl-2 (**D**) proteins in the Hp of mice subjected to CRS. All data were analyzed by two-way ANOVA and Newman–Keuls multiple comparison test. Values are expressed as mean ± SEM; *n* = 4–6. (**E**–**H**) Representative Western blots.

No significant changes were observed in caspase-3, Bid, and Bax protein levels in the PFC (Figure 5A–D) of mice subjected to CRS. However, the level of the Bcl-2 protein in the PFC decreased significantly decreased (Figure 5D, *p* < 0.05). Furthermore, none of the drugs altered the levels of any of the proteins in the Hp (Figure 6A–D). The representative Western blots are shown in Figure 5E–H (PFC) and Figure 6E–H (Hp).

Two-way ANOVA in the PFC for caspase 3: no stress effect [F (1.37) = 0.1237; *p* = 0.7271], no treatment effect [F (3.37) = 0.9085; *p* = 0.4463] and no effect of interaction [F (3.37) = 2.056; *p* = 0.1228]; Bid: no stress effect [F (1.37) = 0.1810; *p* = 0.6730], no treatment effect [F (3.37) = 0.7458; *p* = 0.7458] and no interaction [F (3.37) = 2.192; *p* = 0.1054]; Bax: no stress effect [F (1.37) = 0.1801; *p* = 0.6737], effect of treatment [F (3.37) = 2.431; *p* = 0.0805] and no interaction [F (3.37) = 0.4180; *p* = 0.7411] for Bcl-2 no stress effect [F (1.35) = 0.1318; *p* = 0.2588], no treatment effect [F (3.35) = 2.322; *p* = 0.0920] and effect of interaction [F (3.35) = 4.427; *p* = 0.0097].

Two-way ANOVA in the Hp for caspase 3: no stress effect [F (1.56) = 0.6739; *p* = 0.4152], no treatment effect [F (3.56) = 0.7041; *p* = 0.5536] and no interaction [F (3.56) = 0.1356; *p* = 0.9384]; bid: no stress effect [F (1.56) = 0.5912; *p* = 0.454], no treatment effect [F (3.53) = 2.350; *p* = 0.0829] and no interaction [F (3.53) = 0.4459; *p* = 0.7212]; Bax: no stress effect [F (1.56) = 0.03295; *p* = 0.8566], no treatment effect [F (3.56) = 1.154; *p* = 0.356] and no interaction [F (3.56) = 1.014; *p* = 0.3935]; Bcl-2: no stress effect [F (1.40) = 0.4194; *p* = 0.5210], no treatment effect [F (3.40) = 0.7962; *p* = 0.5578; *p* = 0.6460].

### 2.7. Supplementation with Zn and FLU Treatment in the CRS Model in Mice: Effect on Pro-and Anti-Inflammatory Cytokines.

**Figure 7:**
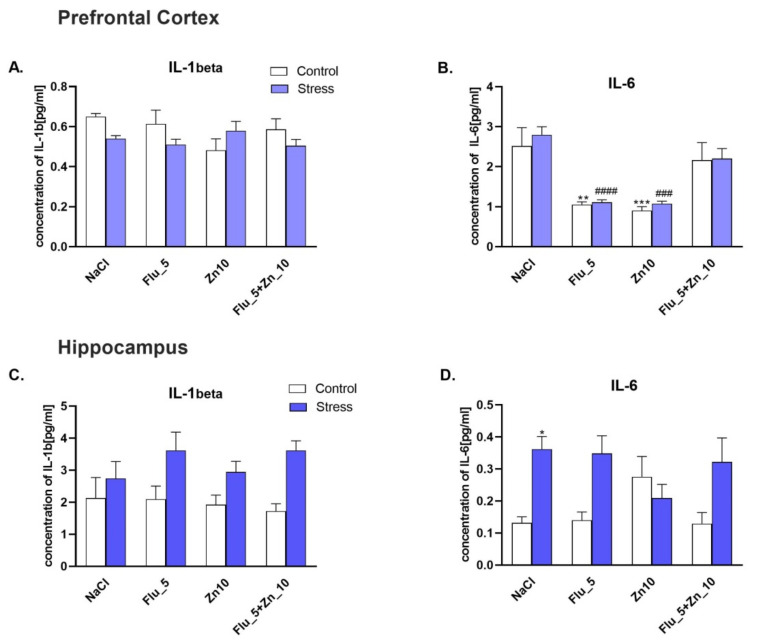
The effect of Zn supplementation and FLU treatment on the concentration of IL-1beta (**A**, **C**) and IL-6 (**B**, **D**) in the PFC and Hp of mice subjected to CRS. All data were analyzed by two-way ANOVA and Newman–Keuls multiple comparison test. Values are expressed as mean ± SEM; *n* = 6–8. * *p* < 0.05; ** *p* < 0.01; *** *p* < 0.001 vs. NaCl; ### *p* < 0.001; #### *p* < 0.0001 vs. CRS + NaCl.

Next, we studied the effects of CRS, Zn, and FLU on the level of interleukins in the PFC (Figure 7A,B) and Hp (Figure 7C,D) of mice. In the PFC, CRS did not influence the level of IL-6, but FLU (5 mg/kg) and zinc (10 mg/kg) given alone decreased the level of IL-6 (Figure 7B). We observed an increased level of IL-6 in Hp (*p* <0.05), however, CRS, Zn, FLU and Zn+FLU did not influence the level of IL-1 beta interleukin.

Two-way ANOVA in the prefrontal cortex for IL-6 showed: no stress effect [F (1.47) = 0.6283; *p* = 0.4319], effect of treatment [F (3.47) = 21.73; *p* < 0.0001] and no interaction [F (3.47) = 0.09937; *p* = 0.9600]; for IL-1b: no stress effect [F (1.47) = 2.438; *p* = 0.125], no treatment effect [F (3.47) = 0.7603; *p* = 0.5220] and no interaction [F (3.47) = 2.495; *p* = 0.0713]. Two way ANOVA in the Hp: for IL-6 showed: stress effect [F (1.54) = 18.11; *p* < 0.0001], no treatment effect [F (3.54) = 0.0816; *p* = 0.9697] and effect of interaction [F (3.54) = 4.314; *p* = 0.0085]; for IL-1b showed: stress effect [F (1.54) = 17.53; *p* = 0.0001], no treatment effect [F (3.54) = 0.4616; *p* = 0.7103] and no interaction [F (3.54) = 0.8455; *p* = 0.4750].

### 2.8. The Combined Effect of Zn Supplementation and FLU Treatment in the CRS Model–Effect on the Level of HDAC Protein Levels.

**Figure 8:**
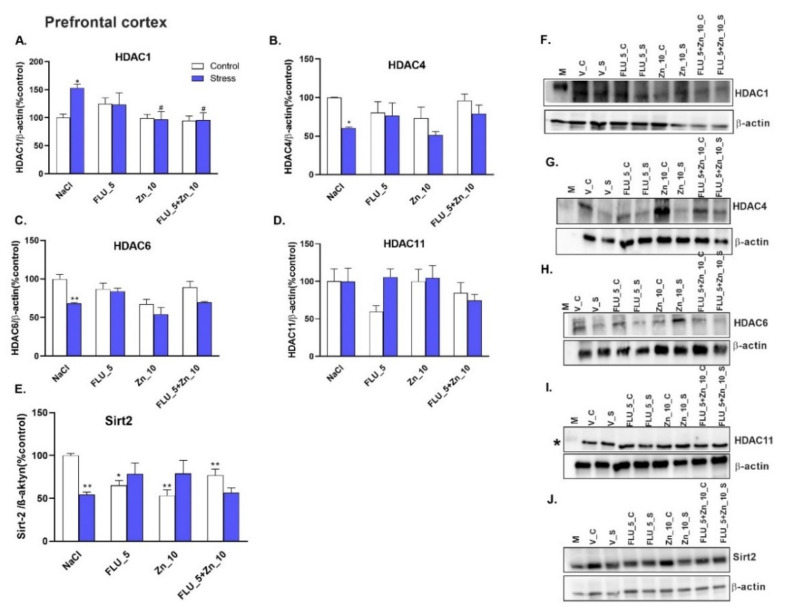
The combined effect of Zn supplementation and FLU treatment on protein levels of HADC1 (**A**), HADC4 (**B**), HDAC6 (**C**), HDAC 11 (**D**), and Sirt2 (**E**) in the PFC of CRS-treated mice. All data were analyzed by two-way ANOVA and Newman–Keuls multiple comparison test. Values are expressed as mean ± SEM; *n* = 4–7. *p < 0.05, **p < 0.01 vs. NaCl; #*p* < 0.05 vs. CRS + NaCl. (F–J) Representative Western blots. * images from the same membrane (see Figure 5).

**Figure 9:**
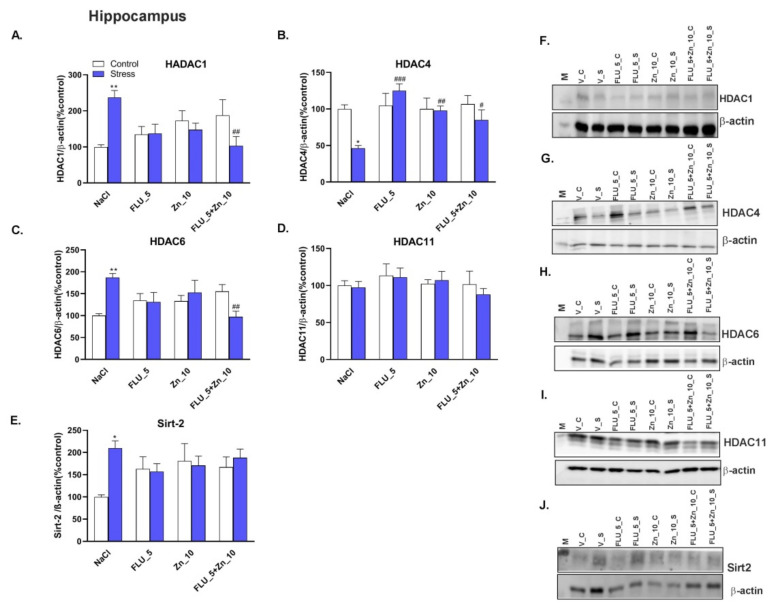
The combined effect of Zn supplementation and FLU treatment on the levels of protein of HADC1 (**A**), HADC4 (**B**), HDAC6 (**C**), HDAC 11 (**D**), and Sirt2 (E) in the Hp of CRS-treated mice. All data were analyzed by two-way ANOVA and Newman–Keuls multiple comparison test. Values are expressed as mean ± SEM; *n* = 8. *p < 0.05, **p < 0.01 vs. NaCl, #*p* < 0.05, ##*p* < 0.01, ###*p* < 0.001 vs. CRS + NaCl. (**F**–**J**) Representative Western blots.

CRS increased the level of HDAC1 in the PFC (Figure 8A) and Hp (Figure 9A) of mice (*p* < 0.05 and *p* < 0.01 respectively), while decreased HDAC4 (*p* < 0.05) both in the PFC (Figure 8B) and Hp (Figure 9B). HDAC6 (*p* < 0.01) and Sirt2 (*p* < 0.01) were decreased in the PFC of mice subjected to CRS (Figure 8C,E) but increased in Hp (Figure 9C,E). Chronic co-administration of FLU (5 mg/kg) with Zn (10 mg/kg) normalized the HDAC1 protein level in the PFC (Figure 8A) and HADC1, HADC4, and HADC6 in the Hp (Figure 9A–C). FLU (5 mg/kg), Zn (10 mg/kg), and FLU (5 mg/kg) + Zn (10 mg/kg) treatments decreased level of Sirt2 in the PFC of control mice (Figure 8E). Representative western blots are shown in Figure 8F–J and Figure 9F–J.

Two-way ANOVA in the PFC for HDAC1 showed: no stress effect [F (1.38) = 2.383; *p* = 0.1310], treatment effect [F (3.38) = 4.103; *p* = 0.0129] and no interaction effect [F (3.38) = 2.820; *p* = 0.0518]; for HDAC4 showed: stress effect [F (1.36) = 7.369; *p* = 0.0101], no treatment effect [F (3.36) = 1.889; *p* = 0.1489] and no interaction [F (3.36) = 1.068; *p* = 0.3748]; for HDAC6 showed: stress effect [F (1.36) = 16.77; *p* < 0.0002], treatment effect [F (3.36) = 7.315; *p* = 0.0006] and no interaction effect [F (3.36) = 2.259; *p* = 0.0982]; for HDAC11 showed: no stress effect [F (1.36) = 0.8852; *p* = 0.3531], no treatment effect [F (3.36) = 1.165; *p* = 0.3364] and no interaction effect [F (3.36) = 1.315; *p* = 0.2845]; for Sirt2 showed: no stress effect [F (1.39) = 1.380; *p* = 0.2471], no treatment effect [F (3.39) = 0.7885; *p* = 0.5076] and interaction effect [F (3.39) = 7.911; *p* = 0.0003].

Two-way ANOVA in the Hp for HDAC1 showed: no stress effect [F (1.56) = 0.1749; *p* = 0.6774], no treatment effect [F (3.56) = 0.6540; *p* = 0.5838] and interaction effect [F (3.56) = 6.687; *p* = 0.0006]; for HDAC4 showed: no stress effect [F (1.56) = 3.243; *p* = 0.0771], treatment effect [F (3.56) = 4.708; *p* = 0.0053] and interaction effect [F (3.56) = 3.935; *p* = 0.0128]; for HDAC6 showed: no stress effect [F (1.56) = 0.9291; *p* = 0.393], no treatment effect [F (3.56) = 0.5051; *p* = 0.803] and interaction effect [F (3.56) = 6.618; *p* = 0.0007]; for HDAC11 showed: no stress effect [F (1.56) = 0.1783; *p* = 0.6745], no treatment effect [F (3.56) = 0.8715; *p* = 0.4614] and no interaction effect [F (3.56) = 0.2238; *p* = 0.8795]; for Sirt2 showed: no stress effect [F (1.56) = 3.140; *p* = 0.0818], no treatment effect [F (3.56) = 0.4930; *p* = 0.6886] and interaction effect [F (3.56) = 2.942; *p* = 0.0408.

## 3. Discussion

Many different procedures have been described in the literature for the CRS animal model of depression. Generally, immobilization for 2–6 h/once daily /10–28 days was found to induce depressive- and anxiety-like symptoms in rodents [18,23,40,41]. Our laboratory adopted 3–6 h of daily immobilization for 28 days. To evaluate CRS-induced depressive-like symptoms, the TST and ST were used. TST is the most commonly used behavioral test for screening potential antidepressant drugs. However, it is also the standard tool for despair testing [42,43]. One of the symptoms accompanying depression is apathy, defined as a lack of interest in different aspects of life, including normal daily tasks and social activities [44]. In rodents, signs of apathy include impaired self-care measured by the ST. ST consists of sprinkling a 10% sucrose solution on a mouse’s coat in its home cage. The viscosity of sucrose solution imitates dirt on the rodent fur and induces grooming behavior. The delay in time between sprinkling and the initiation of grooming and decreased grooming frequency are considered reduced motivational and self-care behaviors associated with the depression-like phenotype [42].

The most prominent depressive effects in our study were observed with the 3-h daily immobilization. Therefore, the 3 h/day/28 days protocol of CRS was chosen for further experiments. The next task was to verify that the depression-like changes induced by CRS will be normalized by treatment with an antidepressant drug. Fluoxetine (FLU), a selective serotonin reuptake inhibitor (SSRI), decreased immobility times in the TST and FST in mice following chronic and acute administration [45]. Chronic treatment with FLU also decreased immobility time in the TST and latency to eat in the novelty-induced hypophagia (NIH) test in adult female mice [46]. Moreover, chronic (21 days) FLU administration reduced depression-like behavior in the social interaction test and FST in the CRS model in mice [47]. Our results mirror those described above. Additionally, in our model, FLU at 10 mg/kg dose reversed all CRS-induced behavioral deficits observed in the TST and ST.

As mentioned above, zinc induced an antidepressant-like effect in the FST and TST in mice [34]. Zinc was also active in different models of depression, such as olfactory bulbectomy (OB), CMS, and CUS. Furthermore, Zn was also shown to improve the effects of standard antidepressants (imipramine, fluoxetine, paroxetine, bupropion, or citalopram) in the TST and CRS in mice and FST and CUS in rats [48,49]. To find out whether Zn supplementation may improve the effect of FLU, a lower dose of FLU (5 mg/kg) was administered jointly with Zn (10 mg/kg) chronically for 14 days. No synergistic effect of Zn + FLU was observed in the TST. All Zn, FLU, and Zn + FLU treatments decreased the CRS-induced increased immobility time in mice. However, the beneficial effect of Zn supplementation was observed in the splash test. Zn + FLU treatment normalized the CRS-induced increased latency to start grooming and extended the amount and time of grooming. This result may suggest that zinc supplementation can improve the antidepressant efficacy of FLU treatment in patients suffering from depression and apathy.

One of the mechanisms of antidepressant action is the modulation of brain-derived neurotrophic factor (BDNF) [50,51,52], a common feature of antidepressant drug action [53]. Our earlier studies showed that the CUS-induced reduction in the BDNF gene expression was antagonized by chronic treatment with zinc [54]. Furthermore, chronic treatment with zinc induced an increase in the BDNF mRNA and protein level in the hippocampus of rats [37]. Finally, our recent studies showed that acute co-administration of IMI and Zn induced antidepressant-like activity in the FST in mice, and this effect correlated with an increase in the level of BDNF protein in the PFC [55]. The modulation of BDNF expression is also associated with the antidepressant action of FLU [56,57]. In the present study, we found that CRS decreased BDNF protein levels in the PFC and Hp of mice; however, the reversal of these changes by FLU, Zn, and Zn + FLU was observed only in the PFC with a trend towards normalization in the Hp. These results further confirm that the mechanism of antidepressant action of Zn and FLU is associated with the modulation of BDNF signaling. However, no synergistic effect of Zn and FLU was seen in the increased BDNF levels.

Inflammation, oxidative stress, and apoptosis are important factors in the pathophysiology of depression and stress-related disorders [58,59,60,61]. The apoptotic process is programmed and controlled by the balance between pro- (Bax) and anti- (Bcl-2) apoptotic proteins within the cell [62]. Data on Bcl-2 and Bax protein levels under restraint stress are inconsistent. In mice, immobility (30 min/28 days) does not change the Bcl-2 protein level; however, the level of caspase-3 increased [63]. Mice stressed for 2 h daily for 21 days (CRS) exhibited an increased Bax/Bcl-2 ratio, caspase-3, IL-1β, and TNF-α in the PFC and Hp [64]. Finally, mice immobilized for 1.5 hr for 7 days showed decreased caspases-3 and no changes in Bcl-2 and Bax protein levels [65]. The only effect observed in our model is the decreased level of the Bcl-2 protein in the PFC, suggesting that CRS-induced apoptotic-like effects are dependent on the duration of stress. However, induction of the antiapoptotic processes is not associated with the antidepressant-like action of FLU + Zn seen in our experiments. The combined administration of Zn and FLU did not cause changes in the level of any of the proteins studied.

There is now ample evidence that the levels of pro-inflammatory cytokines (IL-1β, IL-6, or TNF-α) are elevated in depressed patients and animal models of depression [9,10,66,67]. Interleukins 6 and 1β are inflammatory cytokines, with the former touted as a biomarker for depression [68]. It was found that administration of FLU decreased IL-6 levels in the brain and serum, whereas FLU treatment in the CUMS model decreased IL-6 protein concentration and mRNA level in the Hp [69]. In vitro, Zn (10 and 30 µM) in cell culture decreased TNF-α and IL-6 after LPS administration [70]. We showed that IL-1beta concentration was unchanged in the PFC and Hp, but CRS induced up-regulation of IL-6 in the Hp. Conversely, IL-6 concentration in the PFC decreased following treatment with FLU and Zn alone in control and stressed mice. To summarize this section of our work, CRS for 3 h/28 days induced an inflammatory response in the Hp but not PFC. Zn + FLU treatment does not induce anti-inflammatory effects in the CRS model.

Our data suggest a CRS-induced modulation of deacetylation. CRS increased the levels of HDAC1 protein in the PFC and HDAC1, HDAC6, and Sirt2 proteins in the Hp. However, HDAC4, HDAC6, and Sirt2 protein levels decreased in the PFC and HDAC4 in the Hp. Several works document the level of HDACs in patients with depressive disorders. Peripheral white blood cells of MDD patients exhibited significantly elevated HDAC2 and 5 [71,72] and decreased Sirt1, 2, and 6 protein levels [73]. On the other hand, in the depressive phase in BD patients, increased HDAC4 and decreased HDAC6, 8, and Sirt 1, 2, and 6 protein levels were noted [71,73]. Interestingly, Convington et al. (2009) found decreased levels of HDAC2 in the nucleus accumbens of depressed humans suggesting that peripheral changes in HDAC levels may contradict changes in the brain [74]. Similarly, results from different brain regions in animal models of depression seem contradictory. Most of the available data are related to the social or mild stress procedure. Generally, in these animal models of depression, HDAC2 and HDAC5 were elevated in the Hp but decreased in the nucleus accumbens [27,75]. In the young adult offspring of mice subjected to the gestational stress model, elevated HDAC1 and HDAC2 were correlated with decreased BDNF levels [30]. It is thus not surprising that CRS also decreased the BDNF protein level in our study. Han et al. (2014) confirmed a decrease in HDAC2 and HDAC5 protein levels, but not HDAC 1, 3, and 4 in the Hp after CRS (2 h/14 days) [76]. We showed that HADAC1 increased, but HDAC4 decreased in the Hp and PFC after CRS. HDAC6 is regulated by stress and is elevated in neurodegenerative disorders [77]. Fukada et al. (2012) found that Hdac6 deficient mice exhibit less anxiety in the EPM test and antidepressant-like behavior in the TST, while administration of the Hdac6-specific inhibitor replicated antidepressant-like behavior in mice [77]. This study was confirmed by [78], in which CF (cystic fibrosis) mice with a deletion of HDAC6 restored wild-type growth and inflammatory phenotype. Moreover, decreased levels of HDAC6 impacted mice behavior such that they exhibited anti-depressive properties in the tail suspension test [78]. Our study showed elevated HDAC6 in the Hp of mice subjected to CRS with a decrease in the PFC. The decrease in HDAC6 (and also HDAC2) gene expression in the Hp was also observed by Martin et al. (2017) using CSDS [79].

Sirt2 plays a role in mitochondrial stress and the inflammatory response. In the VGLUT1+/- model of depression, Sirt2 was elevated, and its inhibitor 33i acted as an antidepressant in the sucrose preference test (Munoz-Cobo et al., 2017). Another study with 33i confirmed that inhibition of Sirt2 reversed anhedonia and social avoidance in chronic mild stress [80]. Zhang et al., 2018 found that a lack of Sirt2 blocked the development of stress-induced depressive behavior in social defeat [81]. However, in the CUS model of depression, Sirt2 expression in the Hp decreased and was reversed by fluoxetine treatment [82]. Other authors also confirmed a decrease in hippocampal Sirt2 expression in mice subjected to the CUS and CRS procedures [83]. In contrast, our study showed an increase in Sirt2 protein level in the Hp and a decrease in the PFC. The observed discrepancies may be due to the different stress schedules (2 h/day/14 days vs. 3 h/day/28 days) in both studies. Sirt2 is a member of the HDACIII family and plays a vital role in controlling cell cycle progression, genome stability, and autophagy in neuronal cells. Sirt2 inhibits the transcriptional activation of p53/TP53 and regulates oxidative stress [84]. The increased level of Sirt-2 in the Hp of mice subjected to CRS and the elevated level of inflammatory IL-6 indicated that CRS indeed induced some inflammatory and oxidative stress processes and that this effect is brain region-specific. Chronic treatment with Zn, FLU and Zn + FLU decreased Sirt2 levels in the PFC of naïve mice, indicating that epigenetic modulations might also underlie the antidepressant effect of compounds studied.

The only mechanism that seems to be specific for the effects of Zn + FLU treatment in the CRS model is the modulation of HDACs. Co-administration of FLU + Zn normalized CRS-induced increases in HDAC1 in the PFC and HDAC1, 4, and 6 protein levels in the Hp. In addition, FLU and Zn alone normalized HADC4 levels in the Hp. Furthermore, in the PFC, zinc (not FLU) restored the protein level of HDAC1.

Studies with Balb/c mice indicated that histone deacetylase (HDAC) inhibition enhances the efficacy of FLU. More precisely, treatment with FLU and a class I HDACs (1 and 3) inhibitor elicited antidepressant effects; additional inhibition of class II HDACs was necessary for the anxiolytic effects of FLU. Moreover, the same studies showed that treatment with fluoxetine and various HDAC inhibitors led to significantly enhanced enrichment of acH4K12 at the Bdnf gene promoter 3 and increased expression of Bdnf transcript variant 3 [85]. We can only speculate that the increase in BDNF protein level observed after Zn + FLU treatment might be due to decreased HDAC1 in the PFC. Further studies, e.g., assays measuring the catalytic activity of HADCs are needed to fully unravel the involvement of the modulation of HADCs in the mechanism of the antidepressant-like activity of Zn + FLU treatment.

Despite the different results obtained with the different stress models, CRS in our paradigm appears to be independent of apoptosis but induces inflammation. Despite the limitation mentioned above, our studies suggested that epigenetic processes might underlie the CRS-induced depressive-like effects and antidepressant-like activity of combined Zn + FLU treatment. Our data also indicated that the effects induced both by CRS and Zn + FLU treatment differ between brain structures.

## 4. Materials and Methods

### 4.1. Animals and Drugs Administration

Experiments were carried out on male C57BL/6J mice (~25 g; Charles River, Germany). The animals were kept in a natural day/night cycle at room temperature (21 to 23 °C), with free access to food and water (excluding the restraint period). The mice were housed in groups. After a 1-week acclimatization period to laboratory conditions, mice from stressed groups were subjected to the CRS procedure (3 or 6 h of immobilization/4 weeks) and then to one-day treatment with fluoxetine hydrochloride (5 or 10 mg/kg; i.p.; Tocris) and/or zinc hydroaspartate (10 mg/kg; i.p.; Farmapol) for 14 days. Control groups (CON) received 0.9% NaCl. The injections were carried out between 8:00 a.m. and 2:00 p.m. To verify CRS-induced depressive-like behavior and influence of FLU/FLU + Zn treatment, behavioral tests were performed. At the end of the experiment, the mice were sacrificed to isolate biological material for further research.

### 4.2. Chronic Restraint Stress Procedure

Mice were divided into two groups: Control (not stressed) and stress (subjected to CRS procedure). Control mice were kept in home cages in separate rooms (they did not have contact with stressed animals). Stressed mice were restrained for 3 (8 a.m. to 11 a.m.) or 6 (8 a.m. to 2 p.m.) hours daily for four consecutive weeks using 50 mL tubes enabling easy breathing during the entire period of stress. At the end of each stress session, mice were returned to home cages with unlimited access to food and water. The same steps were repeated every day for 4 weeks.

### 4.3. Splash Test

ST was performed as previously reported [86,87,88]. Each mouse was placed in a clean cage during the test session, and a 10% sucrose solution was spread on the dorsal coat of the animal. Three different parameters were monitored: latency, grooming times; and grooming frequency during a 5-min test period. ST was carried out in a dark room and the cages were lit with a 100 lm bulb. After the test, the mice were returned to their home cages.

### 4.4. Tail Suspension Test

The current study protocol was adapted from [89]. The mice were left hanging from the tip of the tail attached to the flat surface with medical adhesive tape, 30 cm above the floor. The experiment was carried out in dark rooms; otherwise, the rooms were lit centrally by a 100-lumen bulb. The total measurement time was 6 min for each mouse. During this period immobility (defined as freezing and hanging passively without movement) time was measured.

### 4.5. Locomotor Activity

Locomotor activity was measured using an Opto-Varimex-4 Auto-Track (Columbus Instruments, Ohio, USA). The movements of the mice were analyzed in transparent cages (3) covered with lids, a set of four infrared emitters with 16 laser beams each, and four detector tracking and monitoring [90]. Movement detection was analyzed for each mouse separately for 6 min, which is correlated with the TST test. The results were presented as the arithmetic average distance traveled by animals ± SEM for each group.

### 4.6. Western Blot Analysis

Western blot analyzes were performed according to [91]. For Western blot, brain structures such as Hp and the PFC were used. The brains were dissected 24 h after the last drug injection and stored at −80 °C. The total protein level was determined by the BCA method (Pierce BCA Protein Assay Kit (Thermo Scientific, Rockford, IL, USA). Thirty micrograms of protein samples were loaded onto polyacrylamide gels with 2% SDS and sample buffer (Thermo Scientific Pierce Lane Marker Reducing Sample Buffer). Proteins present in the polyacrylamide gels were transferred to a nitrocellulose membrane, blocked in 1% blocking solution (BM Chemiluminescence Western Blotting Kit Mouse/Rabbit Roche) for 1 h, and incubated with specific primary antibodies overnight in 4 °C [rabbit anti- Bcl-2 (D17C4), rabbit anti-Bax (D3R2M), rabbit anti-Caspase-3, rabbit anti-Bid (Cell Signaling); rabbit anti-BDNF (N-20), mouse anti-HDAC1 (H-11), mouse anti-HDAC4 (A-4), mouse anti-HDAC6 (D-11), mouse anti-HDAC11 (C-5), mouse anti-Sirt2 (A-5) Santa Cruz Biotechnology]. After incubation, the membrane was washed 3 times in TBST for 10 min and incubated with secondary antibodies: anti-mouse IgG-peroxidase-conjugated anti-rabbit IgG-peroxidase-conjugated antibodies [diluted 1: 7,000; Roche kit (Roche, Mannheim, Germany) for 1 h at room temperature. Before detection, the membrane was washed 3 times for 10 min in TBST, then read on Fuji-Las 1000 system and measured using a Fuji Image Gauge v.4.0. software (FujiFilm, Tokyo, Japan). The proteins were compared to the optical density of β-actin (mouse anti-β-actin antibody Sigma Aldrich (USA)), 1:10,000) run on the same gel.

### 4.7. ELISA Assays

Levels of interleukin IL-1β and IL-6 were measured by commercial ELISA kits (mouse RayBioTech IL-1β and IL-6). Hp and PFC were sonicated in 2% SDS and used in appropriate protocols. The homogenate was diluted 5× and added to the wells, and incubated for 2.5 h. After incubation and washing, the biotinylated antibody was added. The streptavidin solution was prepared according to the manufacturer’s protocol (ref). The plates were read immediately read at 450 nm. The IL-1β and IL-6 concentrations were calculated based on the total protein level in each probe.

### 4.8. Statistical Analysis

All values are expressed as mean ± standard error of the mean (SEM). The data were analyzed by repeated or factorial analysis of variance one-way ANOVA, followed by post hoc Bonferroni′s or Dunnett′s multiple comparisons tests and two-way ANOVA followed by post hoc Tukey multiple comparisons tests (main CRS experiment and Western blot, ELISA analysis). Statistical analysis was performed using the GraphPad Prism software ver. 8. (GraphPad, San Diego, CA, USA) *p* < 0.05 was considered statistically significant.

## Figures and Tables

**Table 1:** Effect of CRS, FLU, or FLU + Zn treatment on the locomotor activity of mice.

Groups/Treatment	Activity Counts (6-min Test)
A. Control	395.5 ± 19.0
Stress 3 h	382.6 ± 23.0
Stress 6 h	343.9 ± 16.1
B.	
Vehicle	245.1 ± 30.3
Fluoxetine 10 mg/kg	258.0 ± 15.5
CRS + Veh	336.1 ± 17.4
CRS + Fluoxetine 10 mg/kg	241.2 ± 15.6
C.	
Vehicle	397.7 ±31.0
Fluoxetine 5 mg/kg	361.7 ± 28.2
Zinc 5 mg/kg	412.7 ± 25.8
Fluoxetine 5 mg/kg + Zinc 10 mg/kg	314.8 ± 29.6
CRS + Vehicle	296.3 ± 29.6
CRS + Fluoxetine 5 mg/kg	402.3 ± 34.0
Zinc 5 mg/kg	273.5 ± 22.4
Fluoxetine 5 mg/kg + Zinc 10 mg/kg	302.1 ± 15.3

The values represent the mean ± SEM (*n* = 8–13 mice per group) and were evaluated by one (**A**) or two-way ANOVA (**B**, **C**) followed by the Newman–Keuls multiple comparison test.

## Data Availability

Data that support the findings of this study are available from the corresponding author upon reasonable request.

## References

[1] Dean J., Keshavan M. (2017). The neurobiology of depression: An integrated view. Asian J. Psychiatr..

[2] Blackburn T.P. (2019). Depressive disorders: Treatment failures and poor prognosis over the last 50 years. Pharmacol. Res. Perspect..

[3] Herve M., Bergon A., Le Guisquet A.M., Leman S., Consoloni J.L., Fernandez-Nunez N., Lefebvre M.N., El-Hage W., Belzeaux R., Belzung C. (2017). Translational Identification of Transcriptional Signatures of Major Depression and Antidepressant Response. Front. Mol. Neurosci..

[4] Kim Y.K., Won E. (2017). The influence of stress on neuroinflammation and alterations in brain structure and function in major depressive disorder. Behav. Brain. Res..

[5] Sippel L.M., Allington C.E., Pietrzak R.H., Harpaz-Rotem I., Mayes L.C., Olff M. (2017). Oxytocin and Stress-related Disorders: Neurobiological Mechanisms and Treatment Opportunities. Chronic Stress.

[6] Slavich G.M., Irwin M.R. (2014). From stress to inflammation and major depressive disorder: A social signal transduction theory of depression. Psychol. Bull..

[7] Kim Y.K., Na K.S., Shin K.H., Jung H.Y., Choi S.H., Kim J.B. (2007). Cytokine imbalance in the pathophysiology of major depressive disorder. Prog. Neuropsychopharmacol. Biol. Psychiatry.

[8] Siwek M., Sowa-Kucma M., Dudek D., Styczen K., Szewczyk B., Kotarska K., Misztakk P., Pilc A., Wolak M., Nowak G. (2013). Oxidative stress markers in affective disorders. Pharmacol. Rep..

[9] Sowa-Kucma M., Styczen K., Siwek M., Misztak P., Nowak R.J., Dudek D., Rybakowski J.K., Nowak G., Maes M. (2018). Lipid Peroxidation and Immune Biomarkers Are Associated with Major Depression and Its Phenotypes, Including Treatment-Resistant Depression and Melancholia. Neurotox. Res..

[10] Sowa-Kucma M., Styczen K., Siwek M., Misztak P., Nowak R.J., Dudek D., Rybakowski J.K., Nowak G., Maes M. (2018). Are there differences in lipid peroxidation and immune biomarkers between major depression and bipolar disorder: Effects of melancholia, atypical depression, severity of illness, episode number, suicidal ideation and prior suicide attempts. Prog. Neuropsychopharmacol. Biol. Psychiatry.

[11] Erburu M., Munoz-Cobo I., Dominguez-Andres J., Beltran E., Suzuki T., Mai A., Valente S., Puerta E., Tordera R.M. (2015). Chronic stress and antidepressant induced changes in Hdac5 and Sirt2 affect synaptic plasticity. Eur. Neuropsychopharmacol..

[12] Sun X.P., Li S.D., Shi Z., Li T.F., Pan R.L., Chang Q., Qin C., Liu X.M. (2013). Antidepressant-like effects and memory enhancement of a herbal formula in mice exposed to chronic mild stress. Neurosci. Bull..

[13] Xia B., Chen C., Zhang H., Xue W., Tang J., Tao W., Wu R., Ren L., Wang W., Chen G. (2016). Chronic stress prior to pregnancy potentiated long-lasting postpartum depressive-like behavior, regulated by Akt-mTOR signaling in the hippocampus. Sci. Rep..

[14] Varghese F.P., Brown E.S. (2001). The Hypothalamic-Pituitary-Adrenal Axis in Major Depressive Disorder: A Brief Primer for Primary Care Physicians. Prim. Care Companion. J. Clin. Psychiatry.

[15] Campos A.C., Ferreira F.R., Guimaraes F.S., Lemos J.I. (2010). Facilitation of endocannabinoid effects in the ventral hippocampus modulates anxiety-like behaviors depending on previous stress experience. Neuroscience.

[16] Tan S., Wang Y., Chen K., Long Z., Zou J. (2017). Ketamine Alleviates Depressive-Like Behaviors via Down-Regulating Inflammatory Cytokines Induced by Chronic Restraint Stress in Mice. Biol. Pharm. Bull..

[17] Di Liberto V., Frinchi M., Verdi V., Vitale A., Plescia F., Cannizzaro C., Massenti M.F., Belluardo N., Mudo G. (2017). Anxiolytic effects of muscarinic acetylcholine receptors agonist oxotremorine in chronically stressed rats and related changes in BDNF and FGF2 levels in the hippocampus and prefrontal cortex. Psychopharmacology.

[18] Jangra A., Sriram C.S., Dwivedi S., Gurjar S.S., Hussain M.I., Borah P., Lahkar M. (2017). Sodium Phenylbutyrate and Edaravone Abrogate Chronic Restraint Stress-Induced Behavioral Deficits: Implication of Oxido-Nitrosative, Endoplasmic Reticulum Stress Cascade, and Neuroinflammation. Cell Mol. Neurobiol..

[19] Chiu P.Y., Wang C.W., Tsai C.T., Li S.H., Lin C.L., Lai T.J. (2017). Depression in dementia with Lewy bodies: A comparison with Alzheimer’s disease. PLoS One.

[20] Padovan C.M., Guimaraes F.S. (2000). Restraint-induced hypoactivity in an elevated plus-maze. Braz. J. Med. Biol. Res..

[21] Torrisi S.A., Lavanco G., Maurel O.M., Gulisano W., Laudani S., Geraci F., Grasso M., Barbagallo C., Caraci F., Bucolo C. (2021). A novel arousal-based individual screening reveals susceptibility and resilience to PTSD-like phenotypes in mice. Neurobiol. Stress.

[22] Ihne J.L., Fitzgerald P.J., Hefner K.R., Holmes A. (2012). Pharmacological modulation of stress-induced behavioral changes in the light/dark exploration test in male C57BL/6J mice. Neuropharmacology.

[23] Christiansen S.H., Olesen M.V., Wortwein G., Woldbye D.P. (2011). Fluoxetine reverts chronic restraint stress-induced depression-like behaviour and increases neuropeptide Y and galanin expression in mice. Behav. Brain. Res..

[24] Kubera M., Obuchowicz E., Goehler L., Brzeszcz J., Maes M. (2011). In animal models, psychosocial stress-induced (neuro)inflammation, apoptosis and reduced neurogenesis are associated to the onset of depression. Prog Neuropsychopharmacol. Biol. Psychiatry.

[25] Jeon S.W., Kim Y.K. (2018). The role of neuroinflammation and neurovascular dysfunction in major depressive disorder. J. Inflamm. Res..

[26] Salehpour F., Farajdokht F., Cassano P., Sadigh-Eteghad S., Erfani M., Hamblin M.R., Salimi M.M., Karimi P., Rasta S.H., Mahmoudi J. (2019). Near-infrared photobiomodulation combined with coenzyme Q10 for depression in a mouse model of restraint stress: Reduction in oxidative stress, neuroinflammation, and apoptosis. Brain. Res. Bull..

[27] Misztak P., Panczyszyn-Trzewik P., Sowa-Kucma M. (2018). Histone deacetylases (HDACs) as therapeutic target for depressive disorders. Pharmacol. Rep..

[28] Kv A., Madhana R.M., Js I.C., Lahkar M., Sinha S., Naidu V.G.M. (2018). Antidepressant activity of vorinostat is associated with amelioration of oxidative stress and inflammation in a corticosterone-induced chronic stress model in mice. Behav. Brain. Res..

[29] Ignacio Z.M., Reus G.Z., Abelaira H.M., Maciel A.L., de Moura A.B., Matos D., Demo J.P., da Silva J.B., Gava F.F., Valvassori S.S. (2017). Quetiapine treatment reverses depressive-like behavior and reduces DNA methyltransferase activity induced by maternal deprivation. Behav. Brain. Res..

[30] Zheng Y., Fan W., Zhang X., Dong E. (2016). Gestational stress induces depressive-like and anxiety-like phenotypes through epigenetic regulation of BDNF expression in offspring hippocampus. Epigenetics.

[31] Chuang D.M., Leng Y., Marinova Z., Kim H.J., Chiu C.T. (2009). Multiple roles of HDAC inhibition in neurodegenerative conditions. Trends Neurosci..

[32] Shukla S., Tekwani B.L. (2020). Histone Deacetylases Inhibitors in Neurodegenerative Diseases, Neuroprotection and Neuronal Differentiation. Front. Pharmacol..

[33] Wu Y., Hou F., Wang X., Kong Q., Han X., Bai B. (2016). Aberrant Expression of Histone Deacetylases 4 in Cognitive Disorders: Molecular Mechanisms and a Potential Target. Front. Mol. Neurosci..

[34] Nowak G. (2015). Zinc, future mono/adjunctive therapy for depression: Mechanisms of antidepressant action. Pharmacol. Rep..

[35] Szewczyk B., Kubera M., Nowak G. (2011). The role of zinc in neurodegenerative inflammatory pathways in depression. Prog. Neuropsychopharmacol. Biol. Psychiatry.

[36] Nowak G., Szewczyk B., Wieronska J.M., Branski P., Palucha A., Pilc A., Sadlik K., Piekoszewski W. (2003). Antidepressant-like effects of acute and chronic treatment with zinc in forced swim test and olfactory bulbectomy model in rats. Brain. Res. Bull..

[37] Sowa-Kucma M., Legutko B., Szewczyk B., Novak K., Znojek P., Poleszak E., Papp M., Pilc A., Nowak G. (2008). Antidepressant-like activity of zinc: Further behavioral and molecular evidence. J. Neural. Transm. (Vienna).

[38] Cieslik K., Klenk-Majewska B., Danilczuk Z., Wrobel A., Lupina T., Ossowska G. (2007). Influence of zinc supplementation on imipramine effect in a chronic unpredictable stress (CUS) model in rats. Pharmacol. Rep..

[39] Szewczyk B. (2013). Zinc homeostasis and neurodegenerative disorders. Front. Aging. Neurosci..

[40] Halliwell B. (2011). Free radicals and antioxidants - Quo vadis?. Trends. Pharmacol. Sci..

[41] Masneuf S., Lowery-Gionta E., Colacicco G., Pleil K.E., Li C., Crowley N., Flynn S., Holmes A., Kash T. (2014). Glutamatergic mechanisms associated with stress-induced amygdala excitability and anxiety-related behavior. Neuropharmacology.

[42] Becker M., Pinhasov A., Ornoy A. (2021). Animal Models of Depression: What Can They Teach Us about the Human Disease?. Diagnostics (Basel).

[43] Planchez B., Surget A., Belzung C. (2019). Animal models of major depression: Drawbacks and challenges. J. Neural. Transm. (Vienna).

[44] Ishizaki J., Mimura M. (2011). Dysthymia and apathy: Diagnosis and treatment. Depress. Res. Treat..

[45] Xiao W.Z., Zhou W.H., Ma Q., Cui W.G., Mei Q.Y., Zhao X. (2019). Serotonergically dependent antidepressant-like activity on behavior and stress axis responsivity of acacetin. Pharmacol. Res..

[46] Hodes G.E., Hill-Smith T.E., Lucki I. (2010). Fluoxetine treatment induces dose dependent alterations in depression associated behavior and neural plasticity in female mice. Neurosci. Lett..

[47] Yi E.S., Oh S., Lee J.K., Leem Y.H. (2017). Chronic stress-induced dendritic reorganization and abundance of synaptosomal PKA-dependent CP-AMPA receptor in the basolateral amygdala in a mouse model of depression. Biochem. Biophys. Res. Commun..

[48] Ding Q., Li H., Tian X., Shen Z., Wang X., Mo F., Huang J., Shen H. (2016). Zinc and imipramine reverse the depression-like behavior in mice induced by chronic restraint stress. J. Affect Disord..

[49] Szewczyk B., Szopa A., Serefko A., Poleszak E., Nowak G. (2018). The role of magnesium and zinc in depression: Similarities and differences. Magnes. Res..

[50] Franco J.L., Posser T., Brocardo P.S., Trevisan R., Uliano-Silva M., Gabilan N.H., Santos A.R., Leal R.B., Rodrigues A.L., Farina M. (2008). Involvement of glutathione, ERK1/2 phosphorylation and BDNF expression in the antidepressant-like effect of zinc in rats. Behav. Brain Res..

[51] Nowak G., Legutko B., Szewczyk B., Papp M., Sanak M., Pilc A. (2004). Zinc treatment induces cortical brain-derived neurotrophic factor gene expression. Eur. J. Pharmacol..

[52] Szewczyk B., Pochwat B., Rafalo A., Palucha-Poniewiera A., Domin H., Nowak G. (2015). Activation of mTOR dependent signaling pathway is a necessary mechanism of antidepressant-like activity of zinc. Neuropharmacology.

[53] Duman R.S. (2004). Role of neurotrophic factors in the etiology and treatment of mood disorders. Neuromolecular Med..

[54] Cieslik K., Sowa-Kucma M., Ossowska G., Legutko B., Wolak M., Opoka W., Nowak G. (2011). Chronic unpredictable stress-induced reduction in the hippocampal brain-derived neurotrophic factor (BDNF) gene expression is antagonized by zinc treatment. Pharmacol. Rep..

[55] Rafalo-Ulinska A., Poleszak E., Szopa A., Serefko A., Rogowska M., Sowa I., Wojciak M., Muszynska B., Krakowska A., Gdula-Argasinska J. (2020). Imipramine Influences Body Distribution of Supplemental Zinc Which May Enhance Antidepressant Action. Nutrients.

[56] De Foubert G., Carney S.L., Robinson C.S., Destexhe E.J., Tomlinson R., Hicks C.A., Murray T.K., Gaillard J.P., Deville C., Xhenseval V. (2004). Fluoxetine-induced change in rat brain expression of brain-derived neurotrophic factor varies depending on length of treatment. Neuroscience.

[57] Vinet J., Carra S., Blom J.M., Brunello N., Barden N., Tascedda F. (2004). Chronic treatment with desipramine and fluoxetine modulate BDNF, CaMKKalpha and CaMKKbeta mRNA levels in the hippocampus of transgenic mice expressing antisense RNA against the glucocorticoid receptor. Neuropharmacology.

[58] Alcocer-Gomez E., Ulecia-Moron C., Marin-Aguilar F., Rybkina T., Casas-Barquero N., Ruiz-Cabello J., Ryffel B., Apetoh L., Ghiringhelli F., Bullon P. (2016). Stress-Induced Depressive Behaviors Require a Functional NLRP3 Inflammasome. Mol. Neurobiol..

[59] Iwata M., Ota K.T., Duman R.S. (2013). The inflammasome: Pathways linking psychological stress, depression, and systemic illnesses. Brain Behav. Immun..

[60] Khodamoradi K., Amini-Khoei H., Khosravizadeh Z., Hosseini S.R., Dehpour A.R., Hassanzadeh G. (2019). Oxidative stress, inflammatory reactions and apoptosis mediated the negative effect of chronic stress induced by maternal separation on the reproductive system in male mice. Reprod. Biol..

[61] Schiavone S., Jaquet V., Trabace L., Krause K.H. (2013). Severe life stress and oxidative stress in the brain: From animal models to human pathology. Antioxid. Redox. Signal.

[62] Cory S., Adams J.M. (2002). The Bcl2 family: Regulators of the cellular life-or-death switch. Nat. Rev. Cancer.

[63] Seo H., Park C.H., Choi S., Kim W., Jeon B.D., Ryu S. (2016). Effects of voluntary exercise on apoptosis and cortisol after chronic restraint stress in mice. J. Exerc. Nutrition Biochem..

[64] Banagozar Mohammadi A., Torbati M., Farajdokht F., Sadigh-Eteghad S., Fazljou S.M.B., Vatandoust S.M., Golzari S.E.J., Mahmoudi J. (2019). Sericin alleviates restraint stress induced depressive- and anxiety-like behaviors via modulation of oxidative stress, neuroinflammation and apoptosis in the prefrontal cortex and hippocampus. Brain Res..

[65] Lee J.H., Kim T.J., Kim J.W., Yoon J.S., Kim H.S., Lee K.M. (2016). The Anti-apoptotic Effect of Ghrelin on Restraint Stress-Induced Thymus Atrophy in Mice. Immune. Netw..

[66] Leng L., Zhuang K., Liu Z., Huang C., Gao Y., Chen G., Lin H., Hu Y., Wu D., Shi M. (2018). Menin Deficiency Leads to Depressive-like Behaviors in Mice by Modulating Astrocyte-Mediated Neuroinflammation. Neuron.

[67] Szuster-Ciesielska A., Slotwinska M., Stachura A., Marmurowska-Michalowska H., Dubas-Slemp H., Bojarska-Junak A., Kandefer-Szerszen M. (2008). Accelerated apoptosis of blood leukocytes and oxidative stress in blood of patients with major depression. Prog. Neuropsychopharmacol. Biol. Psychiatry.

[68] Dowlati Y., Herrmann N., Swardfager W., Liu H., Sham L., Reim E.K., Lanctot K.L. (2010). A meta-analysis of cytokines in major depression. Biol. Psychiatry.

[69] Shen Z., Xu Y., Jiang X., Wang Z., Guo Y., Pan W., Hou J. (2019). Avicularin Relieves Depressive-Like Behaviors Induced by Chronic Unpredictable Mild Stress in Mice. Med. Sci. Monit..

[70] Hongxia L., Yuxiao T., Zhilei S., Yan S., Yicui Q., Jiamin S., Xin X., Jianxin Y., Fengfeng M., Hui S. (2019). Zinc inhibited LPS-induced inflammatory responses by upregulating A20 expression in microglia BV2 cells. J. Affect Disord..

[71] Hobara T., Uchida S., Otsuki K., Matsubara T., Funato H., Matsuo K., Suetsugi M., Watanabe Y. (2010). Altered gene expression of histone deacetylases in mood disorder patients. J. Psychiatr. Res..

[72] Iga J., Ueno S., Yamauchi K., Numata S., Kinouchi S., Tayoshi-Shibuya S., Song H., Ohmori T. (2007). Altered HDAC5 and CREB mRNA expressions in the peripheral leukocytes of major depression. Prog. Neuropsychopharmacol. Biol. Psychiatry.

[73] Abe N., Uchida S., Otsuki K., Hobara T., Yamagata H., Higuchi F., Shibata T., Watanabe Y. (2011). Altered sirtuin deacetylase gene expression in patients with a mood disorder. J. Psychiatr. Res..

[74] Covington H.E., Maze I., LaPlant Q.C., Vialou V.F., Ohnishi Y.N., Berton O., Fass D.M., Renthal W., Rush A.J., Wu E.Y. (2009). Antidepressant actions of histone deacetylase inhibitors. J. Neurosci..

[75] Fuchikami M., Yamamoto S., Morinobu S., Okada S., Yamawaki Y., Yamawaki S. (2016). The potential use of histone deacetylase inhibitors in the treatment of depression. Prog. Neuropsychopharmacol. Biol. Psychiatry..

[76] Han A., Sung Y.B., Chung S.Y., Kwon M.S. (2014). Possible additional antidepressant-like mechanism of sodium butyrate: Targeting the hippocampus. Neuropharmacology.

[77] Fukada M., Hanai A., Nakayama A., Suzuki T., Miyata N., Rodriguiz R.M., Wetsel W.C., Yao T.P., Kawaguchi Y. (2012). Loss of deacetylation activity of Hdac6 affects emotional behavior in mice. PLoS ONE.

[78] Corey D.A., Rymut S.M., Kelley T.J. (2020). Alleviation of depression-like behavior in a cystic fibrosis mouse model by Hdac6 depletion. Sci. Rep..

[79] Martin V., Allaili N., Euvrard M., Marday T., Riffaud A., Franc B., Mocaer E., Gabriel C., Fossati P., Lehericy S. (2017). Effect of agomelatine on memory deficits and hippocampal gene expression induced by chronic social defeat stress in mice. Sci. Rep..

[80] Erburu M., Munoz-Cobo I., Diaz-Perdigon T., Mellini P., Suzuki T., Puerta E., Tordera R.M. (2017). SIRT2 inhibition modulate glutamate and serotonin systems in the prefrontal cortex and induces antidepressant-like action. Neuropharmacology.

[81] Zhang Z., Zhang P., Qi G.J., Jiao F.J., Wang Q.Z., Yan J.G., He F., Zhang Q., Lv Z.X., Peng X. (2018). CDK5-mediated phosphorylation of Sirt2 contributes to depressive-like behavior induced by social defeat stress. Biochim. Biophys. Acta. Mol. Basis. Dis..

[82] Liu R., Dang W., Du Y., Zhou Q., Jiao K., Liu Z. (2015). SIRT2 is involved in the modulation of depressive behaviors. Sci. Rep..

[83] Wang S.E., Ko S.Y., Jo S., Jo H.R., Han J., Kim Y.S., Son H. (2019). Downregulation of SIRT2 by Chronic Stress Reduces Expression of Synaptic Plasticity-related Genes through the Upregulation of Ehmt2. Exp. Neurobiol..

[84] Jesko H., Wencel P., Strosznajder R.P., Strosznajder J.B. (2017). Sirtuins and Their Roles in Brain Aging and Neurodegenerative Disorders. Neurochem. Res..

[85] Schmauss C. (2015). An HDAC-dependent epigenetic mechanism that enhances the efficacy of the antidepressant drug fluoxetine. Sci. Rep..

[86] Boulle F., Massart R., Stragier E., Paizanis E., Zaidan L., Marday S., Gabriel C., Mocaer E., Mongeau R., Lanfumey L. (2014). Hippocampal and behavioral dysfunctions in a mouse model of environmental stress: Normalization by agomelatine. Transl. Psychiatry.

[87] David D.J., Samuels B.A., Rainer Q., Wang J.W., Marsteller D., Mendez I., Drew M., Craig D.A., Guiard B.P., Guilloux J.P. (2009). Neurogenesis-dependent and -independent effects of fluoxetine in an animal model of anxiety/depression. Neuron.

[88] Yalcin I., Bohren Y., Waltisperger E., Sage-Ciocca D., Yin J.C., Freund-Mercier M.J., Barrot M. (2011). A time-dependent history of mood disorders in a murine model of neuropathic pain. Biol. Psychiatry.

[89] Can A., Dao D.T., Terrillion C.E., Piantadosi S.C., Bhat S., Gould T.D. (2012). The tail suspension test. J. Vis. Exp..

[90] Szopa A., Bogatko K., Herbet M., Serefko A., Ostrowska M., Wosko S., Swiader K., Szewczyk B., Wlaz A., Skalecki P. (2021). The Interaction of Selective A1 and A2A Adenosine Receptor Antagonists with Magnesium and Zinc Ions in Mice: Behavioural, Biochemical and Molecular Studies. Int. J. Mol. Sci..

[91] Pochwat B., Sowa-Kucma M., Kotarska K., Misztak P., Nowak G., Szewczyk B. (2015). Antidepressant-like activity of magnesium in the olfactory bulbectomy model is associated with the AMPA/BDNF pathway. Psychopharmacology.

